# Distinct Substrate Specificities and Electron-Donating Systems of Fungal Lytic Polysaccharide Monooxygenases

**DOI:** 10.3389/fmicb.2018.01080

**Published:** 2018-05-29

**Authors:** Matthias Frommhagen, Adrie H. Westphal, Willem J. H. van Berkel, Mirjam A. Kabel

**Affiliations:** ^1^Laboratory of Food Chemistry, Wageningen University and Research, Wageningen, Netherlands; ^2^Laboratory of Biochemistry, Wageningen University and Research, Wageningen, Netherlands

**Keywords:** LPMO, lytic polysaccharide monooxygenase, substrate specificity, C1/C4-oxidation, electron donor, reducing agent, oxygen, hydrogen peroxide

## Abstract

Lytic polysaccharide monooxygenases (LPMOs) are powerful enzymes that oxidatively cleave glycosidic bonds in polysaccharides. The ability of these copper enzymes to boost the degradation of lignocellulose has greatly stimulated research efforts and biocatalytic applications within the biorefinery field. Initially found as oxidizing recalcitrant substrates, such as chitin and cellulose, it is now clear that LPMOs cleave a broad range of oligo- and poly-saccharides and make use of various electron-donating systems. Herein, substrate specificities and electron-donating systems of fungal LPMOs are summarized. A closer look at LPMOs as part of the fungal enzyme machinery might provide insights into their role in fungal growth and plant-pathogen interactions to further stimulate the search for novel LPMO applications.

## Introduction

The discovery of lytic polysaccharide monooxygenases (LPMOs), in particular fungal LPMOs, has contributed to a revised concept of lignocellulose biodegradation. As opposed to the hydrolytic mechanism, LPMOs have been shown to oxidatively cleave the β-(1→4)-linked bonds in polysaccharides and, to a certain degree, in oligosaccharides (Vaaje-Kolstad et al., 2010; Agger et al., 2014; Isaksen et al., 2014). This is not only relevant for second-generation biorefinery approaches, but will also lead to a better understanding of fungal enzyme machineries and plant-pathogen interactions (Beeson et al., 2015; Johansen, 2016).

The degradation of lignocellulosic plant biomass by fungal enzymes is challenging due to the rigid structure and architecture of the plant cell wall (PCW). The PCW, which functions as a protective coat, allows the plant to resist abiotic and biotic stress factors, like wind and pathogenic fungi. The main PCW compounds comprise cellulose, hemicellulose, proteins, and phenolic compounds such as lignin, and together they form a complex network. The complex and rigid PCW structure hampers its efficient degradation through fungal-based glycosyl hydrolases, which has provoked the exploration of novel enzymes, such as lytic polysaccharide monooxygenases (LPMOs). These LPMOs have been shown to improve the activity of hydrolytic enzyme cocktails and are now seen as essential for lignocellulosic degradation (Dotsen et al., 2007; Brown et al., 2008; Harris et al., 2010; Xu et al., 2012; Hu et al., 2014).

Currently, LPMOs are classified based on their amino acid sequence as auxiliary activity (AA) families AA9, AA10, AA11, and AA13 in the Carbohydrate Active enzyme database (CAZy) (Lombard et al., 2014). The number of AA families has been extended by the discovery of family AA14 comprising fungal LPMOs (Couturier et al., 2018). Remarkably, LPMOs have also been isolated from insects and were classified as family AA15 (Sabbadin et al., 2018). LPMOs cleave the β-(1→4)-linked glucan chain via the oxidation of the C1- or C4 atom in the presence of electron-donating reducing agents and molecular oxygen or hydrogen peroxide (Vaaje-Kolstad et al., 2010; Phillips et al., 2011; Bissaro et al., 2017). Among the six AA groups, AA9 LPMOs show the largest variation in substrate specificity. About 37 fungal LPMO members of the AA9 family that act toward various (1→4)-linked PCW polysaccharides have been characterized since 2010 (Table 1).

**Table 1:** Characterized AA9 LPMOs and there C1/C4-regioselectivity and substrate specificity^a^.

**Organism**	**Enzyme name**	**Regioselectivity**	**Substrate specificity**	**Key references**
*Aspergillus nidulans*	*AN*3046	C1^b^	Cellulose (PASC) Xyloglucan	Jagadeeswaran et al., 2016
*Collariella virescens* (formerly *Chaetomium virescens)*	*Cv*AA9A	C1/C4	Cellulose (PASC) Soluble gluco-oligosaccharides (DP>2) (C4-oxidation only) Xyloglucan (specific)^c^ Xyloglucan oligosaccharides (DP14-18)(specific)^c^ Mixed linked glucan (specific)^c^ Glucomannan Mannohexaose^d^ Xylohexaose^d^ (C4-oxidation only)	Simmons et al., 2017
*Fusarium graminearum*	*Fg*LPMO9A	C1/C4	Cellulose Xyloglucan Longer xylogluco-oligosaccharides	Nekiunaite et al., 2016
*Gloeophyllum trabeum*	*Gt*LPMO9A-2, LPMOA-2	C1/C4	Cellulose Carboxymethylcellulose, Xyloglucan (unspecific) Glucomannan^d^	Kojima et al., 2016
*Geotrichum candidum (Saccharomycotina)*	*Gc*LPMO9A	C1/C4	Cellulose (PASC) Xyloglucan	Ladevèze et al., 2017
	*Gc*LPMO9B	C1/C4	Cellulose (PASC) Xyloglucan	Ladevèze et al., 2017
*Heterobasidion irregulare*	*Hi*LPMO9B	C1	Cellulose (PASC)	Liu et al., 2018
	*Hi*LPMO9H	C1	Cellulose (PASC)	Liu et al., 2017
	*Hi*LPMO9I	C4	Cellulose (PASC) Glucomannan	Liu et al., 2017
*Lentinus similis*	*Ls*(AA9)A	C4	Cellulose (PASC) Soluble gluco-oligosaccharides (DP>2) (C4-oxidation only) Xyloglucan (specific)^c^ Xyloglucan oligosaccharides (DP14-18)(specific)^c^ Mixed linked glucan (specific)^c^ Glucomannan Mannohexaose^d^ Xylan Xylohexaose^d^ (C4-oxidation only)	Frandsen et al., 2016 Frandsen et al., 2017a Simmons et al., 2017
*Myceliophthora thermophila/ Thermothelomyces thermophila* (formerly *Thielavia heterothallica* and *Chrysosporium lucknowence)*	*Mt*LPMO9A	C1/C4	Cellulose (RAC) Xylan associated to cellulose Xyloglucan^d^ Mixed β-(1→3, 1→4)-linked glucan^d^	Frommhagen et al., 2015 Bulakhov et al., 2016 Frommhagen et al., 2016 Gusakov et al., 2017
	*Mt*LPMO9B	C1	Cellulose (RAC)	Frommhagen et al., 2016 Frommhagen et al., 2017a Frommhagen et al., 2017c
	*Mt*LPMO9C	C4	RAC Xyloglucan^c^ Mixed β-(1→3, 1→4)-linked glucan^d^	Frommhagen et al., 2016 Frommhagen et al., 2017b
	*Mt*LPMO9D, MYCTH_92668, *Mt*PMO3^*^	C1 C1/C4 (Vu et al.)	Cellulose (RAC)	Vu et al., 2014a Frommhagen et al., 2017c Span et al., 2017
	*Mt*LPMO9E	C4	Cellulose (PASC, RAC) Xyloglucan Soluble gluco-oligosaccharides (DP>4)	Frommhagen, 2017 Hangasky et al., 2018
	*Mt*LPMO9F	C4	Cellulose (RAC)	Frommhagen, 2017
	*Mt*LPMO9G	C4	Cellulose (RAC)	Frommhagen, 2017
	*Mt*LPMO9	C1/C4	Cellulose (PASC)	Karnaouri et al., 2017
	MYCTH_112089	C1	Cellulose (PASC)	Vu et al., 2014a
*Neurospora crassa*	NCU00836	C1	Cellulose (PASC)	Vu et al., 2014a
	NCU01050, *Nc*LPMO9D, NCPMO-2, PMO-2	C4	Cellulose (PASC)	Phillips et al., 2011 Li et al., 2012 Vu et al., 2014a Bodenheimer et al., 2017
	NCU01867, LPMO-1867, PMO-01867, GH61-10	C1	Cellulose (PASC)	Kittl et al., 2012 Kracher et al., 2016
	NCU02240, *Nc*LPMO9A, GH61-1	C4	Cellulose (PASC)	Vu et al., 2014a Westereng et al., 2016
	NCU02916, *Nc*LPMO9C, LPMO-02916 PMO-02916, *Nc*GH61-3, GH61-3,	C4	Cellulose (PASC) Xyloglucan (unspecific)^c^ Soluble gluco-oligosaccharides Glucomannan Mixed β-(1→3, 1→4)-linked glucan Carboxymethylcellulose	Kittl et al., 2012 Sygmund et al., 2012 Agger et al., 2014 Eibinger et al., 2014 Forsberg et al., 2014a Isaksen et al., 2014 Vu et al., 2014a Borisova et al., 2015 Müller et al., 2015 Courtade et al., 2016 Kojima et al., 2016 Kracher et al., 2016 Nekiunaite et al., 2016 Westereng et al., 2016 Danneels et al., 2017 Karnaouri et al., 2017 Kracher et al., 2017 Breslmayr et al., 2018 Várnai et al., 2018
	NCU03328, *Nc*LPMO9F, LPMO-03328, PMO-03328, GH61-6	C1	Cellulose (PASC)	Kittl et al., 2012 Eibinger et al., 2014 Vu et al., 2014a Kracher et al., 2016 Eibinger et al., 2017 Breslmayr et al., 2018 Várnai et al., 2018
	NCU07760-His6	C1/C4	Cellulose (PASC)	Vu et al., 2014a
	NCU07898, *Nc*LPMO9M, PMO-3	C1, C1/C4 (Vu et al.)	Cellulose (PASC)	Phillips et al., 2011 Li et al., 2012 Vu et al., 2014a
	NCU08760, NCU08760-His6, LPMO-08760, PMO-08760, Gh61-5	C1	Cellulose (PASC)	Phillips et al., 2011 Kittl et al., 2012 Vu et al., 2014a Kracher et al., 2016
*Pestalotiopsis* sp. NCi6	*Ps*LPMOA	C1/C4	Cellulose (PASC)	Patel et al., 2016
	*Ps*LPMOB	C1/C4	Cellulose (PASC)	Patel et al., 2016
*Phanerochaete chrysosporium*	*Pc*LPMO9D *Pc*GH61D, LPMO9D, GH61D	C1	Cellulose (PASC)	Westereng et al., 2011 Wu et al., 2013 Westereng et al., 2013 Westereng et al., 2015 Bissaro et al., 2016 Bissaro et al., 2017 Danneels et al., 2017 Karnaouri et al., 2017 Vuong et al., 2017
*Podospora anserina*	*Pa*LPMO9A, *Pa*GH61A	C1/C4	Cellulose (PASC)	Bey et al., 2013 Bennati-Granier et al., 2015
	*Pa*LPMO9B *Pa*GH61B, GH61B	C1/C4^e^	Cellulose (PASC)	Bey et al., 2013
	*Pa*LPMO9E	C1	Cellulose (PASC)	Garajova et al., 2016 Chabbert et al., 2017
	*Pa*LPMO9H	C1/C4	Cellulose (PASC only) Soluble gluco-oligosaccharides Xyloglucan Glucomannan Lichenan Mixed β-(1→3, 1→4)-linked glucan CMC (indirectly H_2_O_2_)	Bennati-Granier et al., 2015 Garajova et al., 2016 Fanuel et al., 2017 Villares et al., 2017
*Thermoascus aurantiacus*	*Ta*LPMO9A, *Ta*AA9, *Ta*GH61, *Ta*GH61A, *Tau*GH61A,	C1/C4	Cellulose (PASC)	Harris et al., 2010 Quinlan et al., 2011 Müller et al., 2015 Cannella et al., 2016 Kim et al., 2017 Möllers et al., 2017
*Trichoderma reesei (Hypocreae jecorina)*	*Hj*LPMO9A, *Tr*AA9A *Tr*Cel61A, Cel61A, EGIV	C1/C4	Cellulose (PASC)	Tanghe et al., 2015 Bulakhov et al., 2017 Gusakov et al., 2017 Hansson et al., 2017 Danneels et al., 2017 Pierce et al., 2017a Pierce et al., 2017b Song et al., 2018
*Thielavia terrestris*	*Tt*LPMO9E, *Tt*AA9E, *Tt*GH61E	C1	Cellulose (PASC)	Harris et al., 2010 Westereng et al., 2015 Bulakhov et al., 2016 Cannella et al., 2016 Gusakov et al., 2017 Kim et al., 2017 Möllers et al., 2017

a *Only AA9 LPMOs with experimentally determined C1/C4-regioselectivity and substrate specificity are listed, literature referring to obtained crystal structures of the listed LPMOs are not included; PASC, phosphoric-acid swollen cellulose; RAC, regenerated amorphous cellulose*.

b *Inconclusive, C1 oxidation proposed*.

c *Specific (as a function of the backbone substitution) and unspecific oxidation of the β-(1→4)-linked backbone of xyloglucan*.

d *Minor activity*.

e *C1 only with CDH as reducing agent*.

In this study, we first introduce the main PCW constituents, to facilitate understanding of how and to which extent LPMOs act on these structures according to recent revised concepts. For more details about the structure-function relationships of LPMOs of the different AA families we would like to refer to more specific reviews which have been published recently (Frandsen and Lo Leggio, 2016; Forsberg et al., 2017; Meier et al., 2017; Vaaje-Kolstad et al., 2017). Here, we present an overview of AA9 LPMOs, with the main focus on their reported substrate specificities and regioselectivities. Finally, electron-donors of LPMOs are presented and discussed in relation to their possible physiological role. The substrate- and electron donor acceptance of AA9 LPMOs is intriguingly broad, which is why this will be further discussed in relation to future prospects within the biotechnology field.

## Structural and compositional features of the plant cell wall

The PCW is composed of a middle lamella, a primary and a secondary cell wall. The middle lamella is the first synthesized layer of the PCW and mainly contains pectins (Reiter, 2002). The next layer is the primary cell wall, which is the major part of the PCW in fruits and vegetables (Fischer and Bennett, 1991). The primary cell wall is classified into Type I and Type II, based on the structure. The Type I cell wall is present in dicots and to a certain degree in monocots as well (Carpita and McCann, 2008). This Type I consists of cellulose microfibrils, which are embedded in a network built of xyloglucans and, to a lesser extent, glucuronoarabinoxylans (GAXs), as well as pectins such as homogalacturonan and rhamnogalacturonan I (Carpita and Gibeaut, 1993; Hoffman et al., 2005). Type II cell walls are present in Poaceae and in related monocots. In these Type II cell walls, cellulose is embedded in a network of GAXs and, to a lower extent, pectins, glucomannans, and xyloglucans (Gordon et al., 1985; Carpita and McCann, 2008).

The third layer is the secondary PCW, which represents the main part of the dry matter of lignocellulosic feedstocks. Examples of the latter are grass-like agricultural by-products or hard- and soft-woods. The secondary cell wall comprises cellulose and hemicellulose, such as (acetylated) GAXs in grasses and hard woods or mannans in soft woods (Carpita and McCann, 2008). In addition, this cell wall layer is further fortified by non-(hemi)cellulose compounds such as lignin (Harris and Stone, 2009).

### Cellulose

**Figure 1:**
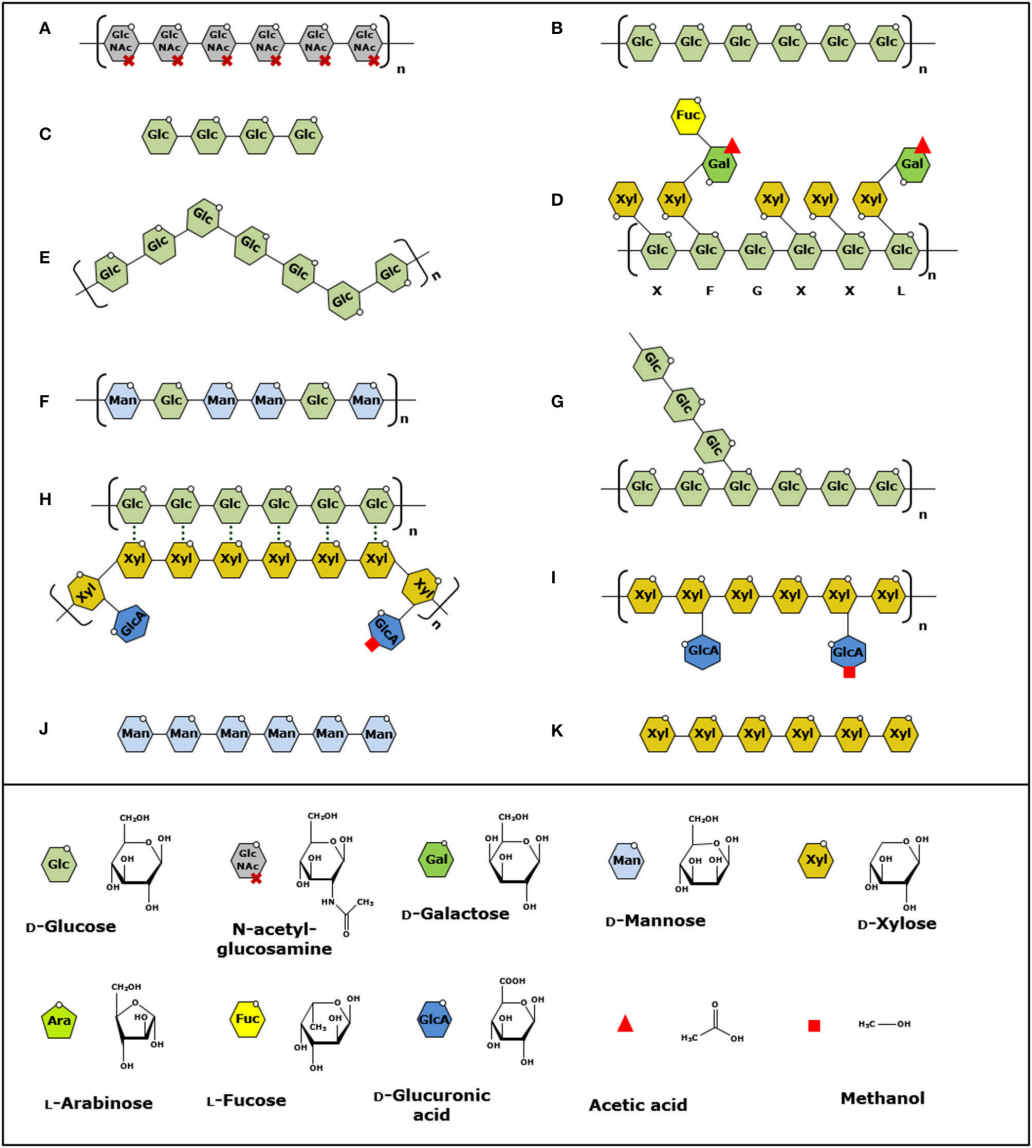
Structural presentation of polysaccharides oxidized by LPMOs. **(A–K)** Order of the polysaccharides is based on the discovery of the LPMO activity toward these substrates. Polysaccharide bonds cleaved by LPMOs are indicated by linkage type. **(A)** β-(1→4)-linked chitin (Vaaje-Kolstad et al., 2010), **(B)** β-(1→4)-linked cellulose (Forsberg et al., 2011), **(C)** soluble β-(1→4)-linked gluco-oligosaccharides (Isaksen et al., 2014), **(D–F)**, β-(1→4)-linked xyloglucan, mixed β-(1→3, 1→4)-linked glucan and β-(1→4)-linked glucomannan (Agger et al., 2014), **(G)** α-(1→4)-linked starch (Vu et al., 2014b), **(H)** β-(1→4)-linked xylan associated to cellulose (Frommhagen et al., 2015), **(I–K)** β-(1→4)-linked xylan, soluble β-(1→4)-linked manno-oligosaccharides and soluble β-(1→4)-linked xylo-oligosaccharides (Simmons et al., 2017). Structural units are shown in the legend on the bottom. Letters under xyloglucan indicate common side chains according to Fry et al. (1993). Structures are modified and based on Scheller et al. (Scheller and Ulvskov, 2010).

Cellulose is a homogeneous linear polymer of β-(1→4)-linked glucan chains which aggregate into microfibrils via hydrogen bonds and van der Waals forces (Nishiyama et al., 2002; Figure 1). The structure of cellulose itself is highly polymorph. Alternate glucosyl units, rotated by 180°, are linked to form a flat ribbon. Hydrogen bonds between the O3-H…O5′ tighten this conformation (Jarvis, 2003). Several of these glucan chains are aligned in parallel and form sheets which are stacked on top of each other with a stagger (Fernandes et al., 2011). The type of stagger depends on the crystal form Iα, which is the predominant form found in algae and bacteria, and Iβ that is present in plants (Atalla and Vanderhart, 1984; Nishiyama et al., 2002, 2003). In addition, these crystalline forms vary in their proportion and type of hydrogen bonds between oxygen atoms, dependent on the source. In general, 12–32 glucan chains form one microfibril, which is twisted and forms a diamond or rectangular shape in higher plants, such as wood (Zhang and Lynd, 2004; Fernandes et al., 2011). The microfibrils differ in length because the glucan chains have either a very high degree of polymerization (DP = 14,000–15,000) in the secondary cell wall (e.g., cotton), or a shorter DP (between 500 and 8,000) in the primary cell wall (Mohnen et al., 2008).

The interaction between the glucan chains and the described packing is not completely coherent within cellulose, which leads to the formation of crystalline and amorphous regions. For example, varying and less strict hydrogen bonds between surface chains of the microfibril allow intermolecular interactions with external molecules in the plant, which were hypothesized to be, for example, hemicelluloses (Viëtor et al., 2002). In addition, pretreatments (e.g., alkaline extraction conditions or heating) can change the structure of cellulose, like its crystallinity (Mohnen et al., 2008; Mittal et al., 2011).

### Hemicellulose

Hemicelluloses are heterogeneous polysaccharides that have different backbones and show structural side-chain variations, like different types and distributions of substituents along the backbone (Figure 1). In addition, backbones of hemicelluloses are strongly associated with cellulose via hydrogen bonding, especially the ones with a low degree of substitution or a blockwise distribution of substituents along the backbone (Vincken et al., 1995; Kabel et al., 2007).

Xylans represent the largest group of hemicellulose in the PCW and can account for up to 50% (w/w) (Ebringerová et al., 2005). All xylans consist of a β-(1→4)-linked xylosyl backbone that can be further substituted by arabinosyl, glucuronosyl, or 4-*O*-methyl-glucuronosyl residues (Darvill et al., 1980; Naran et al., 2008; Gírio et al., 2010). Furthermore, xylans can be covalently bound to lignin via ester and ether linkages through their ferulic and hydrocinnamic acid substitutions (Lam et al., 2001; Hatfield et al., 2008; Kühnel et al., 2012; Figure 1).

Next to xylans, other types of hemicelluloses are found to varying extents in the PCW such as xyloglucans, mixed β-(1→3, 1→4)-linked glucans, mannans, glucomannans, and pectins (Figure 1). Xyloglucans are mostly found in the primary cell wall of higher plants, such as fruit and vegetables or soft- and hard-woods (Park et al., 2004). The backbone of xyloglucan consists of β-(1→4)-linked glycosyl residues that are substituted with xylosyl residues, which are often extended with galactosyl and fucosyl residues (Fry et al., 1993). Mixed β-(1→3, 1→4)-linked glucans are mainly present in the primary cell wall of cereal kernels, such as oat and barley (Scheller and Ulvskov, 2010). In general, three to four β-(1→4)-linked glycosyl units are linked with each other via β-(1→3)-linkages, but longer β-(1→4)-linked segments have also been reported (Bulone et al., 1995; Fry et al., 2008). Mannans and glucomannans mainly consist of β-(1→4)-linked mannosyl units which can be *O*-2 and *O*-3 acetyl-esterified. Next to these mannosyl units, the backbone can also contain glycosyl units as reported for glucomannans and galactomannans (Gilbert et al., 2008). Glucomannans are present in soft- and hard-woods, whereas galactomannans are present mainly in softwoods, such as conifers (Popper and Fry, 2003; Ebringerová et al., 2005).

Pectins are highly heterogeneous polymers, which consist of a plethora of different monosaccharides that show a great variability in linkage type. The three backbone structures are homogalacturonan, xylogalacturonan, and rhamnogalacturonan I, whereas the side chain substructures are arabinan, galactan, and rhamnogalacturonan II (Ridley et al., 2001).

### Lignin and aromatic compounds

Lignin is an aromatic heteropolymer accounting for 10–15% (w/w) of the total dry matter content of grasses and up to 30% of soft- and hard-woods (Rencoret et al., 2009; Del Río et al., 2012). The basic building blocks for the biosynthesis of lignin are the phenylpropane monomers (monolignols) coniferyl, sinapyl, and *p*-coumaryl alcohol. The resulting guaiacyl (G), syringyl (S) and *p*-hydroxyphenyl (H) structural units are linked with each other by C-C and C-O bonds and form a complex 3D-network (Constant et al., 2016). Softwoods mostly comprise lignin with G units, whereas hardwoods have both G and S units (Chen and Dixon, 2007). In contrast, grasses comprise all three structural units G, S, and H. In the PCW, lignin is built into a network with hemicellulose via ester and ether linkages. These linkages are formed between lignin and residues of hemicellulose, which comprise glucuronic acid or arabinosyl-ferulic acid substituents (Takahashi and Koshijima, 1988; Jacquet et al., 1995; Lam et al., 2001).

Apart from lignin, plants contain several other aromatic compounds that are mainly non-cell wall components, which can partly be linked to the PCW. Relevant examples are non-esterified aromatic compounds, like *p*-coumaric acid, ferulic acid, as well as flavonoids such as anthocyanins, which are present in grasses (Cherney et al., 1989). Flavonoids typically consist of two aromatic rings, which are connected via cyclic, aromatic, or aliphatic carbon structures, and have a variety of different substitutions, such as hydroxyl or methoxy groups (Dykes and Rooney, 2007). Other relevant aromatic compounds are tannins, which account for up to 12% (w/w) of the dry matter content in the leaves of some softwoods (Haslam, 2007).

## Discovery of lytic polysaccharide monooxygenases

Due to the PCW complexity a wide range of bacterial and fungal enzymes are involved in the degradation of its PCW polysaccharides. In this respect, enzymes that show hydrolytic activity toward glycosidic bonds are summarized as glycoside hydrolase (GH) families in the CAZy database. Relevant examples are endo- and exoglucanases (GH5, 6, 7, 12, and 45), β-1,4-glucosidases (GH1 and 3) or xylanases (GH10, 11, and 30) (Lombard et al., 2014). GHs interact with their substrates via a pocket, a cleft or a tunnel, which enables them to efficiently degrade hemicelluloses and amorphous regions of cellulose (Davies and Henrissat, 1995; Davies and Williams, 2016). However, in plant biomass, cellulose is mainly present as a crystalline polymer and the surface is hardly accessible for GHs.

**Figure 2:**
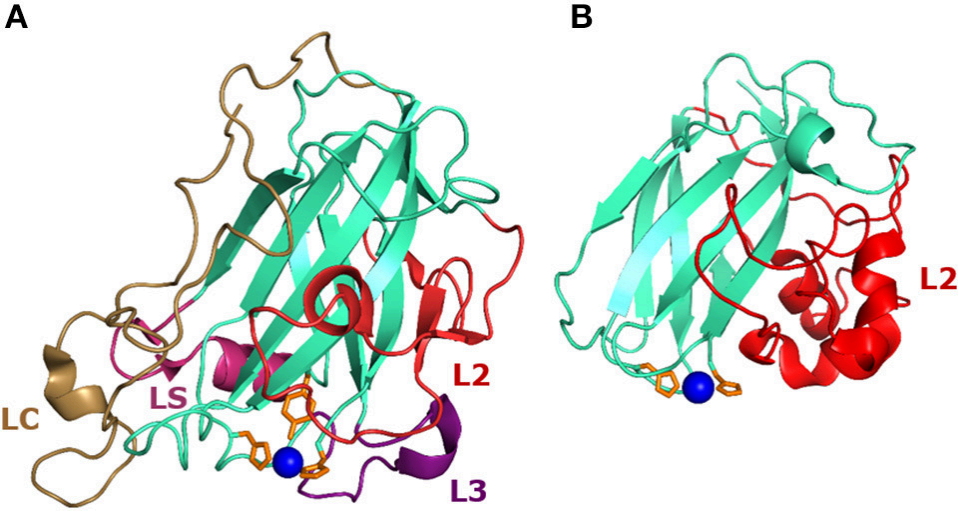
Structure of AA9 and AA10 LPMOs. **(A)** Structure of the *Nc*LPMO9C (PDB-id: 4D7U) from *Neurosspora crassa* which has been the first reported LPMO that oxidizes β-(1→4)-linked hemicelluloses, in addition to β-(1→4)-linked cellulose (Borisova et al., 2015) (Table 1). **(B)** First crystal structure of the chitin-oxidizing CBP21 (*Sm*LPMO19A, PDB-id: 2BEM) from *Serratia marcescens* (Vaaje-Kolstad et al., 2005b). The loops L2, L3, LC, and LS, which are involved in shaping the substrate binding site, are highlighted in red, purple, magenta, and pale brown, respectively. Histidines coordinating the copper ion (blue) are indicated in orange.

Already in 1950, Reese et al. gave first indications of the existence of certain undefined enzymes (“C1”), now considered as LPMOs, that enable cellulolytic bacteria to utilize native cellulose (Reese et al., 1950). In comparison to these cellulolytic prokaryotes, non-cellulolytic bacteria lacked the “C1” enzyme and, therefore, were only able to degrade “shorter linear polyanhydroglucosyl chains.” These results built the foundation of the proposed “C1-Cx-theory” (Reese et al., 1950). A quarter of a century later, Eriksson et al. described an enzyme secreted by *Sporotrichum pulverulentum*, which is able to oxidize cellulose (Eriksson et al., 1974). In addition, the presence of this oxidative enzyme in a cellulolytic enzyme mixture enhanced the cellulose hydrolysis two-fold compared to the hydrolysis of cellulose by a non-oxidative cellulolytic enzyme mixture. Although not further mentioned in detail, the results of the work conducted within the last decade of the past millennium further paved the way toward the discovery of these unique proteins (Raguz et al., 1992; Schrempf and Walter, 1995; Saloheimo et al., 1997). The first crystal structure of such a bacterial-derived protein from *Serratia marcescens* was published by Vaaje-Kolstad et al. (2005b) (Figure 2). In 2010, a breakthrough was achieved with the work of Vaaje-Kolstad et al. who first revealed the oxidative mechanism behind the activity of the bacterial AA10 LPMO, which showed activity toward chitin (Vaaje-Kolstad et al., 2010). This discovery accelerated related research in the field and other LPMOs that oxidized cellulose were soon discovered (Harris et al., 2010; Forsberg et al., 2011; Phillips et al., 2011; Quinlan et al., 2011; Westereng et al., 2011; Figure 2). An overview of substrates known being oxidized by AA9 LPMOs is presented in Figure 1.

The GH61 classification for cellulose active LPMO-like proteins has been first proposed in 1997 (Saloheimo et al., 1997). In comparison, proteins and protein domains with chitin-binding properties were first classified into several carbohydrate-binding module (CBM) families, such as family 33 (CBM33). The CBM33 family mainly comprises bacterial and viral, as well as some eukaryotic non-catalytic chitin-binding proteins (CBPs) (Henrissat and Davies, 1997; Vaaje-Kolstad et al., 2005b; Aachmann et al., 2012). Later, it was recognized that proteins from both GH61 and CBM33 demand electrons for their oxygen-dependent cleavage of polysaccharides, which led to their classification as (lytic) polysaccharide monooxygenases (Phillips et al., 2011; Horn et al., 2012). In 2013, LPMOs were reclassified as auxiliary activity (AA) families (Levasseur et al., 2013; Lombard et al., 2014) and, based on their amino acid sequence similarities, further categorized into AA families 9, 10, 11, 13 and, more recently, 14 and 15 (Cazy.org; Lombard et al., 2014).

## Structural aspects of fungal LPMOs

The first crystal structures of AA9 and AA10 LPMOs were determined before the catalytic activity was revealed in 2010 (Vaaje-Kolstad et al., 2005b, 2010; Karkehabadi et al., 2008). Moreover, structures of AA11, AA13, AA14, and AA15 members have also been published (Hemsworth et al., 2014; Vu et al., 2014b; Couturier et al., 2018; Sabbadin et al., 2018). In general, LPMOs feature a conserved immunoglobulin-like β-sheet core and an extended flat face, which can be enlarged by an α-helical loop (Vaaje-Kolstad et al., 2005b; Harris et al., 2010; Li et al., 2012). The planar surface enables LPMOs to bind to the surface of crystalline polysaccharides, which has been demonstrated by using NMR spectroscopy (Aachmann et al., 2012). By using X-ray crystallography, LPMOs have been identified as monocopper enzymes (Quinlan et al., 2011). The copper ion is coordinated by a N-terminal histidine, a second side chain histidine and, depending on the AA class, a third aromatic amino acid such as tyrosine in case of AA9 LPMOs (Karkehabadi et al., 2008). These residues involved in the copper coordination are forming the so-called “histidine brace” (Hemsworth et al., 2013). More details about the structural features of LPMOs are further highlighted in relation to their substrate specificity, C1-/C4-regioselectivity and catalytic mechanism in the following chapters.

## Substrate specificity and C1/C4-regioselectivity of LPMOs

### Substrate specificity and structure-function relationships

All LPMOs contain multiple extended loops that are involved in polysaccharide recognition and binding via shaping the substrate-binding surface, such as the L2 and L3 loop, the short (LS) and the long C-terminal (LC) loop (Vaaje-Kolstad et al., 2005b; Aachmann et al., 2012; Li et al., 2012; Wu et al., 2013; Borisova et al., 2015; Figure 2). Depending on the substrate specificity, these loops comprise various hydrophilic and aromatic residues that are involved in the substrate binding of LPMOs. For instance, AA9 LPMOs often comprise multiple aromatic amino acids, such as tyrosines, which are expected to take part in the binding of crystalline cellulose (Vaaje-Kolstad et al., 2005b; Forsberg et al., 2011; Li et al., 2012). In contrast to AA9 LPMOs, chitin active LPMOs of the AA10 family feature more hydrophilic amino acids, such as Thr, Gln, or Ser, to bind, compared to cellulose, the more hydrophilic chitin surface (*N*-acetylglucosamine) via polar interactions (Vaaje-Kolstad et al., 2005b; Aachmann et al., 2012; Li et al., 2012).

Until 2013, AA9 LPMOs were known to oxidize insoluble substrates, like chitin, cellulose, and its derivatives (Figure 1). In 2014, Isaksen et al. reported a fungal LPMO (*Nc*LPMO9C) that oxidizes soluble substrates, in particular, soluble cello-oligosaccharides (Isaksen et al., 2014; Figure 1C). A follow-up study described the activity of *Nc*LPMO9C toward hemicellulose structures such as xyloglucan and mixed β-(1→3, 1→4)-linked glucan (Borisova et al., 2015; Figures 1E–G). On this basis, AA9 LPMOs have been further described to cleave xyloglucan independent of the side chains attached to the β-(1→4)-linked glucan backbone like *Fg*LPMO9A and *Gt*LPMO9A-2, whereas others oxidize xyloglucan more specifically, such as *Nc*LPMO9C and *Pa*LPMO9E (Agger et al., 2014; Bennati-Granier et al., 2015; Kojima et al., 2016; Nekiunaite et al., 2016; Fanuel et al., 2017). Next, we demonstrated that, in the presence of cellulose, *Mt*LPMO9A from *M. thermophila* C1 is capable of oxidizing xylan associated to cellulose (Frommhagen et al., 2015; Figure 1H). Moreover, it was shown that the *Cv*AA9A from *Collariella virescens* oxidizes isolated xylan in addition to various hemicelluloses (Simmons et al., 2017) (Figures 1I–K). Recently, the AA14 LPMOs *Pc*AA14A and *Pc*AA14B have been shown to be active toward xylan-coated cellulose fibers, which is indicative that also LPMOs of other AA families may exhibit activity toward was wider range of PCW polysaccharides (Couturier et al., 2018). Similar to xyloglucan, the β-(1→4)-linked xylosyl backbone is also substituted, but it has not been shown yet if the above-mentioned xylan-oxidizing LPMOs show a similar cleavage behavior in the present of side chains as described for xyloglucan-oxidizing LPMOs.

The discovery of LPMOs that act toward soluble polysaccharides, such as *Nc*LPMO9C, *Pa*LPMO9H, *Ls*(AA9)A, *Cv*AA9A, and *Mt*LPMO9E opened new possibilities to investigate the enzyme-substrate interaction by using X-ray crystallography and NMR spectroscopy (Bennati-Granier et al., 2015; Courtade et al., 2016; Frandsen et al., 2016, 2017a; Frommhagen, 2017; Simmons et al., 2017). One of these studies showed that soluble polysaccharides dock to the histidine brace and an extended flat binding surface, which also involves the L3 and LC loop as determined for *Nc*LPMO9C (Courtade et al., 2016). Interestingly, amino acid residues of structurally investigated LPMOs interact with different sugar moieties of the bound substrates. For example, using X-ray crystallography, Frandsen et al. showed that the +1 sugar is stacked to the methylated His-1 of *Ls*(AA9)A and that binding ranges from the −4 to +2 sugar (Frandsen et al., 2016). In contrast, based on NMR spectroscopy, Courtrade et al. suggested that the binding range of *Nc*LPMO9C ranges from the −3 to +3 or −2 to +4 sugar (Courtade et al., 2016). Simmons et al. presented a substrate-depending binding mode of *Ls*AA9A and *Cv*AA9A when they investigated enzyme-substrate complexes using different polysaccharides with various substituents (Simmons et al., 2017). The above-mentioned studies clearly showed that different amino acid residues of the binding surface are involved in substrate binding ranging from residues that build up hydrogen bonds [Asn28, His66, and Asn67 for *Ls*(AA9)A] and aromatic residues, like Tyr204 and Tyr203 in *Nc*LPMO9C and *Ls*(AA9)A, respectively (Courtade et al., 2016; Frandsen et al., 2016; Simmons et al., 2017).

The phylogenetic relationship between substrate specificity, C1/C4-regioselectivity and amino acid sequence has not been identified so far. A major obstacle to understand the structure-function relationships of LPMOs is the large amino acid sequence variability among these enzymes (Lenfant et al., 2017). Moreover, studies have identified thousands of putative AA9-encoding genes in sequenced genomes among the fungal kingdom and secretomics indicated that a large number of these LPMOs are actually secreted if fungi were grown on lignocellulosic biomass (Mahajan and Master, 2010; Grigoriev et al., 2014; Rytioja et al., 2014a; Kracher et al., 2016; Frommhagen et al., 2017a; Lenfant et al., 2017; Mäkelä et al., 2017). It becomes apparent that more LPMO-substrate interactions, especially involving crystalline polysaccharides, have to be uncovered to enhance the understanding of the structure-function relationships of LPMOs and to revise already implemented sub-classifications of AA9 family members (Li et al., 2012; Hemsworth et al., 2013, 2015; Bennati-Granier et al., 2015). Compared to the widely used global multiple sequence alignment, the recently proposed clustering analysis of fungal AA9 LPMOs, which is based on local pairwise alignments, could be an alternative approach to improve the phylogenetic analysis (Lenfant et al., 2017). Possibly, an enhanced understanding of the structure-function relationships of LPMOs may also help to recognize why the substrate binding affinity and the catalytic performance cannot be easily correlated (Forsberg et al., 2014b).

It is likely that the substrate specificity of AA9 LPMOs is further influenced by CBMs that are appended to LPMOs, like members of the CAZy-CBM family 1, 2, and 33 (Gilkes et al., 1991; Lombard et al., 2014). Appended CBMs have already been described to be involved in the substrate recognition and interaction and, thereby, they can influence the activity of carbohydrate active enzymes (Cantarel et al., 2009). However, the impact of appended CBMs on the activity of LPMOs highly depends on the type of substrate or LPMO used (Forsberg et al., 2014b; Borisova et al., 2015; Crouch et al., 2016; Hansson et al., 2017). It has been reported that truncation or replacement of an appended CBM increased, diminished or did not influence the catalytic performance of the LPMO. Depending on the substrate and LPMO, it is likely that the CBM binding nature influences the positioning of a specific LPMO to which the CBM is appended to, since these modules have already been described to exhibit different binding behaviors and preferences toward polysaccharides (Taylor et al., 2012; King et al., 2015; Arola and Linder, 2016). Hereby, the CBM could impact the mode of action of the LPMO by promoting either a random or more processive substrate oxidation, similar to random and processive glycosyl hydrolases.

Notably, LPMOs are secreted proteins that often contain posttranslational modifications, such as multiple disulfide bonds and N-glycosylation sites. Some of the loops, such as the L2 loop, build disulfide bridges with the conserved β-sheet core (Wu et al., 2013; Figure 2A). Since some AA9 members contain 5–10 monosaccharide units, it was suggested that glycosylations in the planar face could potentially impact the substrate binding, as shown for a CBM1 by using molecular dynamics simulations (Li et al., 2012; Taylor et al., 2012). Fungal AA9 LPMOs contain a τ-N-methylated histidine (His-1), which is involved in coordination of the copper cofactor (Quinlan et al., 2011; Li et al., 2012). In contrast, bacterial AA10 LPMOs and AA9 LPMOs produced in “foreign” hosts, such as *Pichia pastoris*, do not contain this τ-N-methylation (Li et al., 2012; Hemsworth et al., 2013; Wu et al., 2013; Bennati-Granier et al., 2015). Recently, Frandsen et al. suggested that the methylation of the τ-N-terminal His1 affects the LPMOs stability due to the altered p*K*a, which makes the imidazole of His1 less likely to be protonated at a lower pH compared to the imidazole of His78 (Frandsen et al., 2017a). Still, the role of this τ-N-methylation remains debatable since some LPMO members either with or without the τ-N-methylation at His1 were active, which led to the conclusion that this posttranslational modification is not strictly necessary to maintain LPMO activity (Westereng et al., 2011; Borisova et al., 2015).

### *In vivo* and *in vitro* aspects of substrate specificity

About 10 AA9 LPMOs have been shown in literature to be active toward hemicellulose or soluble gluco-oligosaccharides (Table 1). Lately, this list has been extended by the discovery of fungal AA14 LPMO that shows activity toward xylan-coated cellulose fibers (Couturier et al., 2018). The discovery of LPMOs, which are active toward hemicellulose structures, led to a paradigm shift in the understanding of PCW degradation. The ability of LPMOs to attack these polysaccharides indicates that these enzymes may have an important function in opening up the lignocellulose matrix to assist subsequent extensive degradation by hydrolytic enzymes. Two examples are xylan and xyloglucan, which are non-covalently associated with cellulose within the PCW (Vincken et al., 1995; Kabel et al., 2007). The planar surface of LPMOs may enable them to oxidize these less accessible regions, which is followed by a disruption of the cellulose and hemicellulose structure and thereby results in an enhanced accessibility for hydrolases.

The expectation is that more and more LPMOs will be discovered showing different substrate specificities. Within the fungal kingdom, certain fungal species contain multiple AA9 LPMO-encoding genes, which have been shown to be expressed differently when the fungi are grown on different polysaccharides (Tian et al., 2009; Vanden Wymelenberg et al., 2009; Berka et al., 2011; Mäkelä et al., 2017). Certainly, absolute numbers of putative LPMO-encoding genes have to be interpreted cautiously since not all of these LPMOs have been shown to actually be present in the fungal secretome (Tian et al., 2009; Mahajan and Master, 2010; Rytioja et al., 2014b; Mäkelä et al., 2017). Still, these numbers are indicative of the fact that the discrimination of LPMOs by their substrate specificity is currently not complete. Some fungi even express multiple putative LPMO-encoding genes to utilize only pure cellulose, such as Avicel (Rytioja et al., 2014a). It is likely that LPMOs are exhibiting further modes of action, similar to glycosyl hydrolases, which can range from random to processive hydrolytic cellulose cleavage (Kurašin and Väljamäe, 2011; Jung et al., 2013; Kubicek et al., 2014). Reported AA9 LPMOs oxidize cellulose but recent findings indicate that cellulose-specificity potentially ranges from the preferred oxidation of highly crystalline to completely amorphous regions (Table 1; Eibinger et al., 2014; Villares et al., 2017). Moreover, LPMOs that are active toward crystalline cellulose may further be distinguished by their preference for microfibrils comprising the Iα or Iβ crystal form, which has yet to be investigated (Nishiyama et al., 2002, 2003; Jarvis, 2003). A similar scenario has already been described for CBMs and some members have been shown to be able to bind hardwood cellulose but not bacterial cellulose (Arola and Linder, 2016; Chabbert et al., 2017; Eibinger et al., 2017). Another example is shown by a recent study in which the cleaving behavior of *Nc*LPMO9F on the cellulose surface is revealed by using time-resolved atomic force microscopy (Eibinger et al., 2014). This LPMO oxidized the surface layer of the crystalline cellulose region only, which resulted in the formation of a patch, but amorphous regions were not oxidized. Moreover, a vertical degradation of subjacent layers mediated by *Nc*LPMO9F was hardly determined. In comparison, another study described the activity of *Pa*LPMO9H, which was only active toward amorphous cellulose, but not toward crystalline cellulose fibers (Villares et al., 2017; Table 1).

### C1/C4-regioselectivity

**Figure 3:**
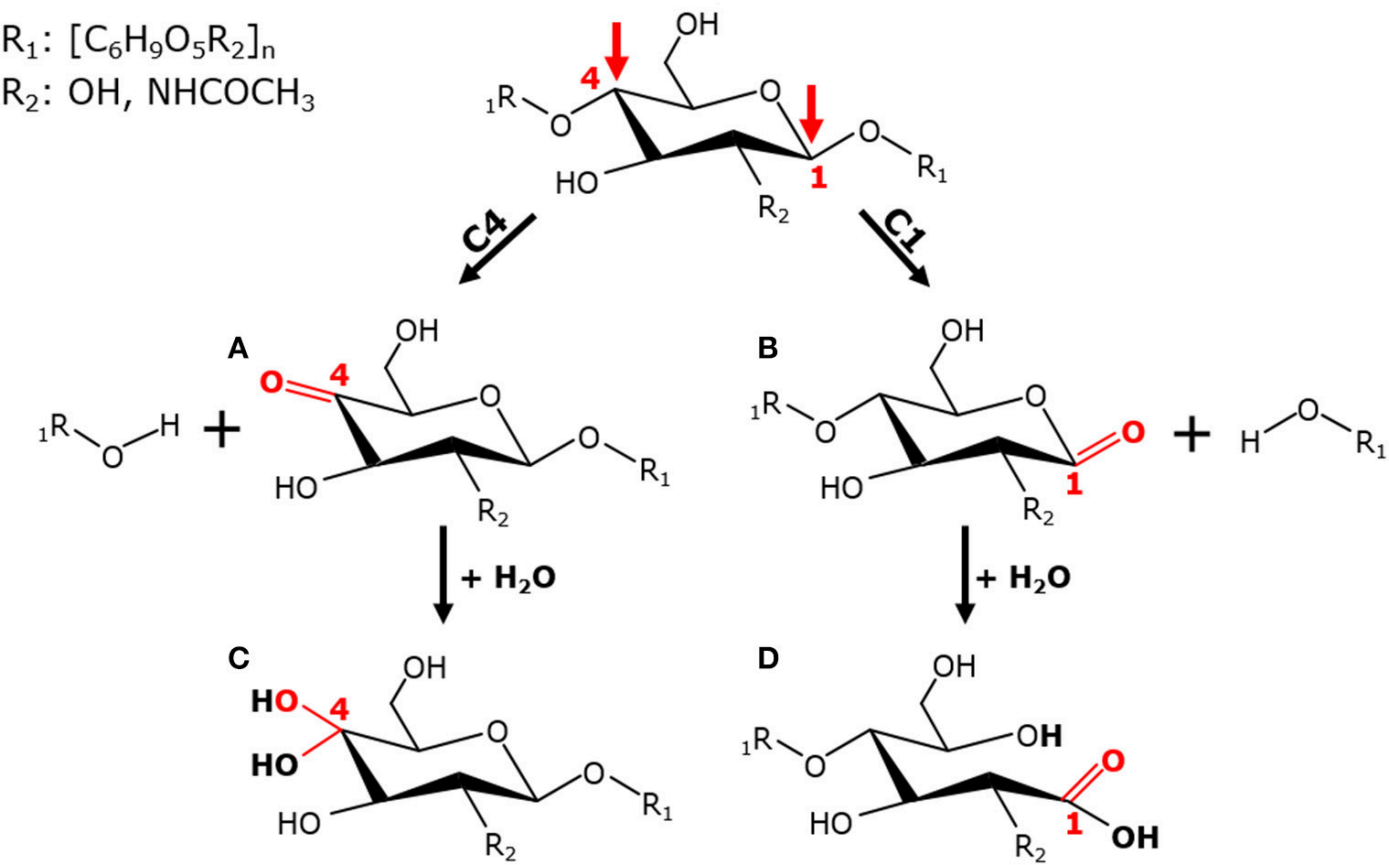
C1/C4-regioselectivity of LPMOs. Oxidation of β-(1→4)-linked glucan at the C1- or C4-carbon position by LPMOs leads to the formation of non-oxidized and C1- or C4-oxidized oligosaccharides. The LPMO-mediated C4-oxidation results in the formation of 4-ketoaldoses **(A)**, which are present as their corresponding hydrates, geminal diols, in aqueous solutions **(C)**. The oxidation at the C1-atom leads to formation of a labile δ-lactone **(B)**, which dissociates in water into an aldonic acid **(D)**.

The first C1/C4-regioselectivities have been experimentally determined for two bacterial LPMOs. Both CBP21 (AA10) from *S. marcescens* and CelS2 (AA10) from *Streptomyces coelicolor* oxidize the C1-carbon position of chitin and cellulose, respectively (Vaaje-Kolstad et al., 2010; Forsberg et al., 2011). This C1-oxidation of the reducing end sugar of chitin or cellulose leads to the formation of an unstable δ-lactone (−2 Da), which is converted into an aldonic acid in the presence of water (+ 16 Da) (Vaaje-Kolstad et al., 2010; Forsberg et al., 2011; Figure 3). Shortly thereafter, the fungal AA9 LPMO *Ta*GH61 was shown to oxidize cellulose at the C1 as well as at the C4 position (Quinlan et al., 2011; Figure 3). The latter leads to the oxidation of the C4-carbon of the non-reducing end sugar, which results in the formation of a more stable 4-ketoaldose (−2 Da) that tautomerizes into a geminal diol depending on the pH (Quinlan et al., 2011; Isaksen et al., 2014; Westereng et al., 2016; Figure 3). From this point onwards, the number of AA9 and AA10 LPMOs with known C1/C4-regioselectivity grew rapidly (Phillips et al., 2011; Westereng et al., 2011). AA9 LPMOs with experimentally determined C1/C4-regioselectivities are summarized in Table 1. It should be noted that more LPMOs have been expressed and characterized, but their C1-/C4-regioselectivity has not been experimentally determined yet.

Until today, AA9 LPMOs have been shown to oxidize either the C1, C4 or both the C1 and C4 position (Table 1). In contrast, bacterial AA10 LPMOs cleave cellulose and chitin by the oxidation of the C1 or both, the C1 and C4 carbon atom, whereas no AA10 member has been published to singly oxidize the C4-position (Forsberg et al., 2011, 2014a, 2017). The described AA11 and AA13 LPMOs, as well as the recently discovered AA14 and AA15 LPMOs, are all C1-oxidizing LPMOs (Hemsworth et al., 2014; Vu et al., 2014b; Lo Leggio et al., 2015; Couturier et al., 2018; Sabbadin et al., 2018).

The reason for the existence of LPMOs with different regioselectivities is not yet fully understood. In fact, it has been shown that a single fungus, such as *Neurospora crassa, Myceliophthora thermophila* C1, or *Podospora anserina*, can express LPMOs that oxidize cellulose either at C1 or C4, or at both carbon positions (Table 1). The C1/C4-regioselectivity is likely the result of how the LPMO binds to the substrate, meaning that different LPMOs may bind to differently exposed cellulose-fiber surfaces. As mentioned earlier, cellulose is structurally polymorph and contains of varying microfibril structures, hydrophobic and hydrophilic sides, a different degree of crystallinity and diverse intra- and intermolecular hydrogen bonds and bridges between oxygen atoms (Atalla and Vanderhart, 1984; Nishiyama et al., 2002, 2003; Jarvis, 2003; Fernandes et al., 2011). The thereby established differences in conformation, shape and compactness of the β-(1→4)-linked glucan chains could limit the accessibility of the C1- or C4-carbon atom, which is a possible explanation for the evolution of LPMOs exhibiting different regioselectivities.

So far, published data addressing a possible relation between structural features of LPMOs and C1/C4-regioselectivity is limited (Frandsen and Lo Leggio, 2016; Vaaje-Kolstad et al., 2017). Borisova et al. showed that strictly C1-oxidizing AA9 LPMOs such as *Tt*LPMO9E contain a conserved tyrosine residue (Tyr80 based on *Nc*LPMO9C), which is Ala80 in the C4-oxidizing *Nc*LPMO9C (Borisova et al., 2015). The presence of this residue likely restricts the accessibility to the solvent-facing carbon atom in the axial coordination position (Borisova et al., 2015). Interestingly, Simmons et al. showed that fungal LPMOs seem to feature a substrate-specific C1/C4-regioselectivity. Both of the LPMOs tested, *Ls*AA9A and *Cv*AA9A, oxidized the C4-carbon of shorter oligosaccharides whereas longer polysaccharides were cleaved via the oxidation of the C1- and C4-carbon atom (Simmons et al., 2017). A more recent study demonstrated the involvement of active-site residues, such as the above-mentioned tyrosine, in the C1/C4-regioselectivity of LPMOs (Forsberg et al., 2017). Mutation of active-site residues of the C1/C4-oxidizing *Ma*LPMO10B from *Micromonospora aurantiaca* let to decrease of the C4-oxidizing activity and loss of chitin specificity, whereas these *Ma*LPMO10B mutants maintained the C1-oxidizing regioselectivity. Moreover, truncation or mutation of residues of the *Ma*LPMO10B-appended CBMII did not alter the C1/C4-regioselectivity of *Ma*LPMO10B (Forsberg et al., 2017). These recent studies illustrate how marginal sequence variations and substrate-binding interactions alter the C1/C4-regioselectivity, which will challenge a further classification of LPMOs based on their experimentally determined substrate specificity and C1/C4-regioselcetivity.

## Catalytic mechanism of LPMOs

Before LPMOs were described as copper-containing oxygenases, isotope labeling (${\text{H}}_{2}^{18}$O and ^18^O_2_) indicated that one of the two oxygens introduced into the oxidized glucan chain derived from water and the other one from molecular oxygen (Vaaje-Kolstad et al., 2010). In addition, it was shown that this LPMO-mediated reaction demanded electrons, which were provided by reducing agents, such as ascorbic acid or reduced glutathione (Vaaje-Kolstad et al., 2010). In 2011, the first detailed reaction pathway was proposed by Phillips et al. who based the catalytic mechanism on the reaction pathway of copper monooxygenases (Klinman, 2006; Phillips et al., 2011; Solomon et al., 2011).

A key step described the one-electron reduction of Cu(II) to Cu(I) by either an external reducing agent or a cellobiose dehydrogenase (CDH) (Phillips et al., 2011). The Cu(I) binds molecular oxygen and forms a copper superoxo intermediate via an internal electron transfer. Based on the proposed mechanism, the copper superoxo intermediate subtracts a hydrogen atom from the C1-carbon or the C4-carbon atom of the polysaccharide, depending on the C1/C4-regioselectivity of the LPMO. The following reactions include a second reduction step and the recovery of the Cu(II) oxidation state, via formed intermediates such as copper hydroperoxo intermediate and a copper oxo radical, and the release of water (Phillips et al., 2011). Upon the end of this reaction, a substrate radical is formed that reacts further with an additional oxygen atom which destabilizes the β-(1→4)-linkage followed by the elimination reaction and the formation of a non-oxidized and an oxidized glucan chain (Phillips et al., 2011; Beeson et al., 2012). It was also suggested that a direct two electron reduction of oxygen to a Cu-OOH intermediate might be able to abstract a hydrogen (Phillips et al., 2011). However, this reaction pathway was not experimentally underpinned since neither did peroxide enable the reaction nor was the reaction inhibited by catalase addition (Phillips et al., 2011). In the following years, more detailed reaction mechanisms have been proposed (Beeson et al., 2015; Walton and Davies, 2016). These reaction pathways share the idea that Cu(II) is reduced by an external electron to Cu(I) at the start of the reaction. However, for the second step it remains debatable whether the Cu(I)-LPMO first binds oxygen or the substrate (Walton and Davies, 2016). The existence of various intermediate steps, such as the copper-dependent oxygen activation or the binding of oxygen and superoxide, are experimentally and computationally supported by using known LPMO structures (Li et al., 2012; Kjaergaard et al., 2014; Frandsen et al., 2016; Bertini et al., 2017). Intriguingly, new catalytic mechanisms have been proposed, which include H_2_O_2_ as a key compound or, dependent on the substrate, facilitate more than one catalytic route (Bissaro et al., 2017; Simmons et al., 2017). Both findings are described below in more detail.

## Mechanism of the electron transfer

Beside the discussions about the reaction mechanisms, there is an ongoing debate about how LPMOs receive electrons from external reducing agents or enzymes. One major point of discussion is that the close binding of LPMOs to the surface of cellulose or chitin makes the catalytic copper center inaccessible, even for smaller reductants (Courtade et al., 2016; Frandsen et al., 2016; Simmons et al., 2017). Therefore, it was hypothesized that the reduction of the catalytic copper center takes place before the binding of the LPMO to the polysaccharide or, alternatively, electrons are continuously transported to the catalytic copper center through electron tunneling. Li et al. suggested two possible scenarios how fungal AA9 LPMOs could accommodate an internal electron tunneling (Li et al., 2012). In the first scenario, electrons are transferred through an internal conserved ionic network (hydrogen bond network), which connects the copper ligand tyrosine with the surface-exposed Lys under involvement of conserved tyrosine residues. In a second scenario, electrons are provided by aromatic residues from the proximity of the histidines (Li et al., 2012). In addition, computational simulation studies showed that the heme domain of a CDH could potentially bind to a hydrophilic conserved surface patch. The electrons could be transferred to the catalytic copper center by one of the above-proposed scenarios (Li et al., 2012). Interestingly, follow-up research strengthened both of the scenarios by providing more experimental evidence that AA9 LPMOs interact with a reducing agent either via a surface patch centered around the Pro-Gly-Pro triad or a narrow patch around the amino acid residues that coordinate the copper ion (Li et al., 2012; Tan et al., 2015; Courtade et al., 2016).

## Catalytic performance of LPMOs as a function of the electron donor

**Figure 4:**
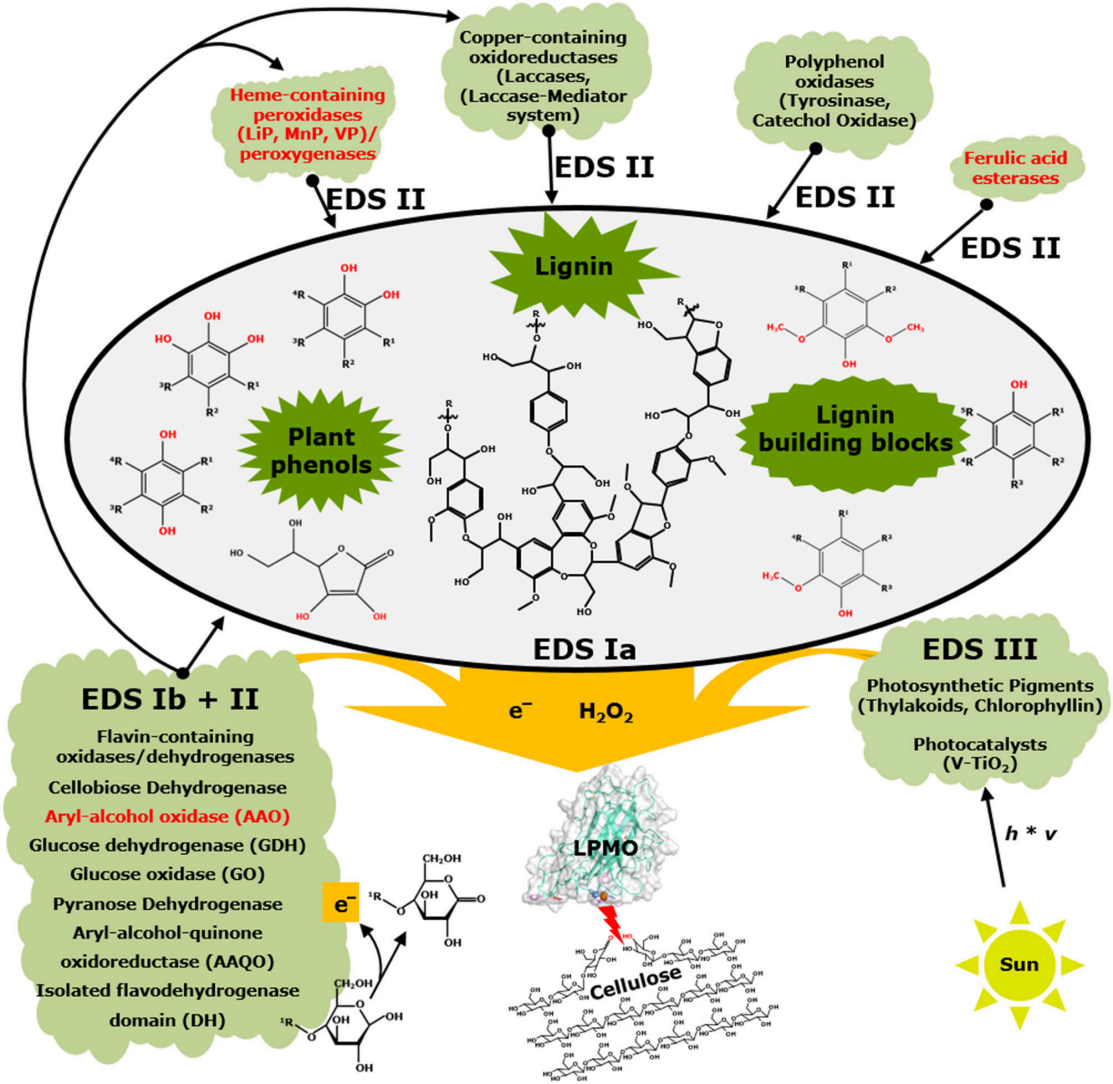
Interactions between non-enzymatic and enzymatic electron-donating systems and their effect on LPMOs. Black fonts represent electron-donating systems Ia and b, II, and III (EDS) that have been published. Red fonts illustrate systems that have not yet been shown to be members of electron-donating systems of LPMOs. This scheme is simplified and co-substrates such as hydrogen peroxide or oxygen as well as enzymes acting on reactive oxygen species (EDS IV) are not included. In addition, aerobic and anaerobic conditions are not further considered. Schematic presentation of lignin is based on Zakzeski et al. (2010). Further details are described in the text.

Interestingly, research has also shown that the reducing agent preference varies among LPMOs (Figure 4). In general, phenolic compounds comprising structural features like the 1,2-dihydroxy (IIa), 1,2-dihydroxy-6-methoxy (IIb), and 1,2,3-trihydroxy moiety (III) efficiently reduce LPMOs (Westereng et al., 2015; Frommhagen et al., 2016; Kracher et al., 2016). In addition, compounds that do not comprise the above-mentioned features have also been shown to reduce LPMOs such as ascorbic acid, glutathione and L-cysteine or the macromolecule lignin (Vaaje-Kolstad et al., 2010; Lo Leggio et al., 2015; Westereng et al., 2015; Figure 4). In comparison, monophenols are less efficient electron donors for LPMOs (Frommhagen et al., 2016). The reducing efficiency of a compound can be determined experimentally by cyclic voltammetry and expressed as the reduction potential. Phenolic compounds comprising a second hydroxyl group attached to the benzene ring have a relatively low reduction potential (≤250 mV), whereas monophenols have a relatively high reduction potential (≥400 mV; Kracher et al., 2016). Kracher et al. (2016) showed that a lower reduction potential of reducing agents results in a higher catalytic performance of LPMOs (Kracher et al., 2016). Hence, it seems possible to predict if a compound is an efficient electron donor for LPMOs, based on its structure and associated reduction potential. But there are exceptions like the *ortho*-diphenol quercetin, which does not promote LPMO activity for three different AA9 LPMOs tested (Frommhagen et al., 2016).

The reduction potential of a number of LPMOs has been determined to be around +250 mV (vs. SHE). However, some members show deviating values which are significantly higher (+326 mV vs. SHE) or lower (+155 mV vs. SHE) (Aachmann et al., 2012; Borisova et al., 2015; Garajova et al., 2016; Kracher et al., 2016). These deviating values may result from differences in the protein structure. Thus, LPMOs comprising a low reduction potential (e.g., below +250 mV (vs. SHE)] likely receive electrons from compounds with a relatively low reduction potential. In contrast, LPMOs with a high reduction potential [e.g., above +250 mV (vs. SHE)] can also receive electrons from reducing agents which comprise a reduction potential that is, for example, around +250 mV (vs. SHE).

Still, the catalytic performance in the presence of various efficient electron donors with a low reduction potential (e.g., compounds comprising a 1,2-dihydroxy moiety) differs vastly among LPMOs. These differences are caused by numerous factors, which have to be carefully considered when interpreting obtained results from LPMO activity assays, such as reducing agent stability or operational stability of the LPMO in the presence of different reducing agents (Frommhagen et al., 2016; Bissaro et al., 2017). Furthermore, the amino acid residues present in the surface patch centered around the Pro-Gly-Pro triad or the narrow patch around the copper ion differ in structure and surface charge distribution among LPMOs (Frommhagen et al., 2016). Therefore, it seems plausible that the interaction between these amino acid residues and reducing agents is different for each of the LPMOs. Notably, the above-mentioned properties such as surface charge of the enzyme or reduction potential are largely influenced by incubation conditions like pH, temperature or the type of buffer used (Frommhagen et al., 2017c). High concentrations of reducing agents, in particular the ones with a low reduction potential, have been reported to inactivate LPMOs within minutes by forming reactive oxygen species (ROS) (Bissaro et al., 2017). Alternatively, the catalytic performance of LPMOs might be influenced by the pH due to alterations in the coordination of the copper ion in the active site, as a harsh decrease in the pH may lead to the protonation of the imidazole (Frandsen et al., 2017a). Such pH-dependent rearrangements of active-site histidines could also affect copper and substrate binding (Frandsen et al., 2017b).

New insight in the reaction mechanism of LPMOs might be brought up by an intriguing recent finding of Bissaro et al. (2017), who showed that H_2_O_2_ is a co-substrate of LPMOs (Bissaro et al., 2017). These authors proposed that reducing agents are directly involved in the “priming reduction” which describes the reduction of LPMO-Cu(II) to LPMO-Cu(I) (Bissaro et al., 2017). In the reduced stage, the LPMO-Cu(I) reacts with H_2_O_2_ and the thereby formed copper-bound oxygen species subtract a hydrogen atom from either the C1- or the C4-carbon atom of the polysaccharide. In a further step, the copper-bound oxygen species reacts with the carbon atom, which leads to the hydroxylation of the substrate and, under molecular rearrangement, to the cleavage of the β-(1→4)-linkage (Bissaro et al., 2017). More recently, quantum mechanical/molecular mechanical (QM/MM) calculations supported the findings of Bissaro and colleagues by demonstrating that LPMOs are able to break the O-O bond of H_2_O_2_ (Wang et al., 2018). The subsequently generated Cu(II)-oxyl species oxidizes the C4-H bond and the thereby formed hydroxylated C4-carbon intermediate leads to spontaneous non-enzymatic chain cleavage (Wang et al., 2018). Hangasky et al. (2018) showed that the C4-oxidizing *Mt*LPMO9E utilizes both O_2_ and H_2_O_2_, dependent on the assay condition. Based on their results, *Mt*LPMO9E oxidized cellulose specifically at the C4-position in the presence of O_2_, whereas H_2_O_2_ addition led to a non-specific polysaccharide oxidation (Hangasky et al., 2018). These results will contribute to the ongoing debate about the role of H_2_O_2_ on the catalytic mechanism of LPMOs and, furthermore, should be considered regarding the determination of the C1/C4-regioselectivity of LPMOs in the presence of varying assay conditions.

Interestingly, LPMOs of family AA9 and AA10 that exhibit the H_2_O_2_-driven peroxygenase-like reaction have a significantly higher catalytic performance compared to LPMOs that are active in the presence of O_2_ (Bissaro et al., 2017; Kuusk et al., 2017). In addition, Bissaro et al. (2017) have shown that reducing agents, such as ascorbic acid, are also able to stimulate the formation of H_2_O_2_ in the absence of AA9 LPMOs (Bissaro et al., 2017). Based on their findings, it is conceivable that more scenarios may impact the reducing agent preference of LPMOs, such as different sensitivities of LPMOs for H_2_O_2_ (Bissaro et al., 2017). Still, the proposed priming reduction which describes the activation of the Cu(II)-LPMO via one electron reduction to the Cu(I)-LPMO demands a direct interaction between reducing agent and LPMO. In addition to Bissaro et al. (2017), Simmons et al. (2017) showed evidence that LPMOs may exhibit a substrate-depending catalytic mechanism, which is based on the observation that different oxygen species may be bound to the copper ion if *Ls*AA9 is bound to cello-oligosaccharides compared to xylo-oligosaccharides (Simmons et al., 2017). They observed that the catalytic performance of *Ls*AA9 toward xylan is less dependent on the type of reducing agent present, which corroborates that LPMOs perform more than one catalytic mechanism. Combining the recent findings, it becomes apparent that several factors, which impact the reducing agent preference of LPMOs, are not fully understood so far.

## Electron-donating systems of LPMOs

In 2010, it became apparent that LPMOs demand electrons from small molecular weight compounds, such as ascorbic acid and gallic acid (Vaaje-Kolstad et al., 2010). Later, other electron-donation systems were discovered, like the co-action of LPMOs with CDH (Phillips et al., 2011). Westereng et al. were the first who reported lignin as a possible electron donor for *Tt*LPMO9E (Westereng et al., 2015). Today, it is generally accepted that LPMOs can receive electrons from multiple types of reducing agents. Therefore, reducing agents published in literature were classified into four different electron-donating systems (EDS I-IV). It should be noted that some members of one electron-donating system (Ib), such as CDH, can also be part of another system (II), and vice versa, dependent on the activity.

### Electron-donating system I

EDS I includes compounds (EDS Ia) and enzymes (EDS Ib) that directly donate electrons to LPMOs. This system includes numerous compounds that are mainly plant phenols and lignin building blocks, such as monophenols and phenolic compounds comprising a 1,2-benzendiol or 1,2,3-benzenetriol moiety, as well as lignin (Westereng et al., 2015; Frommhagen et al., 2016; Kracher et al., 2016; Figure 4). The flavin-containing CDH, which is a member of the GMC (glucose-methanol-choline oxidase/dehydrogenase) oxidoreductase family, was the first enzyme reported to reduce LPMOs directly and, so far, is the most used enzyme system to study the LPMO mechanism *in vitro* (Phillips et al., 2011; Kittl et al., 2012; Sygmund et al., 2012; Bey et al., 2013; Garajova et al., 2016; Figure 4). Phillips et al. (2011) showed that the deletion of the highly expressed CDH-1 gene from the cellulolytic fungus *N. crassa* resulted in a decreased cellulose degradation. Moreover, the addition of a purified CDH from *M. thermophila* to a CDH lacking *N. crassa* strain improved the cellulose degradation up to two-fold (Phillips et al., 2011). In addition, studies have shown that LPMOs and CDH are co-expressed with various growth substrates, which indicates the relevance of this interaction in nature (Hori et al., 2011; Langston et al., 2011; Yakovlev et al., 2012; Navarro et al., 2014; Couturier et al., 2015).

Next to CDH, other GMC members, such as glucose oxidase (GOx), glucose dehydrogenase (GDH), pyranose dehydrogenase (PDH), aryl-alcohol quinone oxidoreductases (AAQO), aryl-alcohol oxidase (AAO), and an isolated flavodehydrogenase domain (DH), have been shown to either donate electrons directly to LPMOs or regenerate electron-donating plant phenols and lignin building blocks (Garajova et al., 2016; Kracher et al., 2016; Bissaro et al., 2017). In that respect some of the GMC members also belong to “Electron-donating system II.” As an example, a GDH from *Glomerella cingulata* added to the incubation with *Nc*LPMO9C (LPMO-02916) in the presence of various quinones improved the oxidation of cellulose (Kracher et al., 2016). In addition, sequence analysis showed a positive correlation between the occurrence of GMC oxidoreductase-encoding genes and LPMO-encoding genes among fungi (Kracher et al., 2016). In another study, a GDH from *Pycnoporus cinnabarinus* was shown to donate electrons to *Pa*LPMO9H (Garajova et al., 2016). In this study two variants of AAQOs (AAQO1 and AAQO2) from *P. cinnabarinus* were described that can be used to reduce *Pa*LPMO9E. Until now, aryl-alcohol oxidases (AAOs) have not been shown to influence the catalytic performance of LPMOs, such as the AAO from *Ustilago maydis*, which did not reduce *Pa*LPMO9E (Garajova et al., 2016). Moreover, some GMC family members (e.g., AAO, CDH) are reported to interact either via electron donation or H_2_O_2_ formation with other enzymes, such as laccases and peroxidases, which may affect the catalytic performance of LPMO indirectly (Temp and Eggert, 1999; Hildén et al., 2000; Hernández-Ortega et al., 2012; Figure 4). Next to flavin-dependent oxidoreductases, a pyrroloquinoline quinone (PQQ)-dependent pyranose dehydrogenase (PDH) from *Coprinopsis cinerea, Cc*PDH, has been shown to reduce both the C1-oxidizing *Nc*LPMO9F and C4-oxidizing *Nc*LPMO9C from *N. crassa* (Várnai et al., 2018).

### Electron-donating system II

EDS II comprises enzymes that release and modify reducing agents that influence the activity of LPMOs. Recently, polyphenol oxidases (PPOs), such as *Mt*PPO7 from *M. thermophila* C1 and *Ab*PPOs from *Agaricus bisporus*, have been shown to affect the activity of LPMOs indirectly by their monophenolase and diphenolase activity (Frommhagen et al., 2017a; Figure 4). More specifically, the polyphenol oxidase *Mt*PPO7 from *M. thermophila* C1 converts methoxylated phenolic compounds that are present as lignin-building blocks in the PCW into compounds comprising a 1,2-dihydroxy moiety and, thereby, enhances the catalytic performance of *Mt*LPMO9B. Sequence analysis of genomes of 336 Ascomycota and 208 Basidiomycota revealed a high correlation between genes encoding *Mt*PPO7-like proteins and AA9 LPMOs, which is indicative for the coupled action of PPOs and LPMOs in the concerted degradation of lignocellulosic biomass (Frommhagen et al., 2017a). Peroxidases such as manganese peroxidases (MnPs), lignin peroxidases (LiPs), and versatile peroxidases (VPs) as well as laccases are known to oxidize plant phenols, lignin building blocks and lignin and, therefore, are also likely to effect the electron donation of these compounds toward LPMOs (Hammel et al., 1993; Hofrichter, 2002; Camarero et al., 2005; Pérez-Boada et al., 2005; Figure 4). Moreover, it has been demonstrated that the laccase-mediator system (LMS) can be used to partially break down lignin into small-molecular-weight compounds which are potential electron donors for LPMOs (Brenelli et al., 2018).

Presumably, other reducing agent releasing and/or modifying enzymes, which take part in PCW degradation, will also affect the activity of LPMOs, such as ferulic acid esterases (Figure 4). Ferulic acid esterases have been shown to release coumaric acid and ferulic acid from PCW hemicelluloses, like in grains and grasses (Kühnel et al., 2012; Várnai et al., 2014). As described above, these cinnamic acid derivatives have been shown to be hydroxylated by PPOs into compounds comprising 1,2-dihydroxy moieties, which are efficient electron donors for LPMOs (Frommhagen et al., 2017a).

### Electron-donating system III

EDS III includes light-induced non-enzymatic systems. In 2016, Cannella et al. (2016) reported that light in combination with photosynthetic pigments, such as thylakoids or chlorophillin, are very efficient electron-donating systems for the reduction of LPMOs (Cannella et al., 2016; Figure 4). Next to these pigments, chemical photocatalysts like vanadium-doped titanium dioxide (V-TiO2) can also efficiently reduce LPMOs (Bissaro et al., 2016; Figure 4). Above all, these systems comprise the advantage of a switch-on/off possibility and, therefore, are a useful tool for investigating the catalytic mechanism of LPMOs (Bissaro et al., 2016, 2017; Cannella et al., 2016; Möllers et al., 2017).

### Electron-donating system IV

EDS IV comprises enzymes that alter superoxide or hydrogen peroxide levels to affect the catalytic performance of LPMOs. Catalase and superoxide dismutase can affect the LPMO activity or stability in an indirect way due to altering superoxide or hydrogen peroxide concentrations during the incubation (Scott et al., 2016; Bissaro et al., 2017). As an example, the catalase from *Thermoascus aurantiacus* (Accession DD046677) reportedly decreases the inactivation of AA9 LPMOs during biomass degradation (Scott et al., 2016). Apart from that, both superoxide dismutase and catalase may also be involved in most of the non-enzymatic and enzymatic electron-donating systems, since superoxide and hydrogen peroxide are released during the oxidation/reduction (redox) reactions (Bissaro et al., 2017). Furthermore, the hydrogen peroxide generating xanthine oxidase (XOD) has been shown to reduce *Sc*LPMO10C, which led to the oxidation of cellulose (Bissaro et al., 2017). The same AA10 LPMO was also active toward cellulose in the presence of chemical superoxide generating systems, such as potassium superoxide (KO_2_) (Bissaro et al., 2017).

## Electron-donating systems *in vivo*

So far, most LPMO electron-donating systems have been determined *in vitro*. In general, LPMOs used for the degradation of lignocellulosic biomass are secreted enzymes, which oxidize polysaccharides outside of the fungal cell. The physiological relevance of LPMO electron-donating systems is likely to be determined by the mode of life-style that fungi exhibit.

For instance, saprophytic fungi such as the Ascomycota *M. thermophila* C1 and *N. crassa* or the Basidiomycota *Phanerochaete chrysosporium*, which comprise already multiple characterized AA9 LPMOs (Table 1), mainly feed on dead biomass that often contains high amounts of lignin and other phenolic compounds, next to polysaccharides (Martinez et al., 2004; Phillips et al., 2011; Frommhagen et al., 2017a). For these saprophytic fungi, LPMOs are likely to receive electrons from lignin and lignin-building blocks (Figure 4). Presumably, LPMOs secreted by these fungi benefit from other secreted oxidative enzymes during the concerted degradation of biomass, such as the GMC oxidoreductases, PPOs and, although not shown yet, laccases and peroxidases (Phillips et al., 2011; Kracher et al., 2016; Frommhagen et al., 2017a; Figure 4). It is unlikely that the light-induced non-enzymatic electron-donating system will act as an efficient electron donor for LPMOs, which are secreted by saprophytic fungi. The latter are able to decompose biomass in the absence of light (e.g., in the soil or ground level of a forest) or degrade parts of the plant that do not contain photosynthetic pigments (e.g., the stamp of hardwoods). Also intracellular reducing agents, such as ascorbic acid and reduced glutathione, are not expected to play a major role as reducing agents for LPMOs, since dead biomass will not comprise intact cells that contain these rather instable compounds.

In contrast to saprophytes, pathogenic fungi are known to attack the PCW of living cells such as the rice pathogen *Magnaporthe grisea* or the wheat pathogen *Fusarium graminearum*, which both comprise multiple putative AA9 LPMO-encoding genes (Dean et al., 2005; Nekiunaite et al., 2016; Frommhagen et al., 2017a). LPMOs secreted by these pathogenic fungi are possibly used to disrupt the cell wall in order to improve the penetration of the fungus into the plant cell. Thereby, LPMOs are likely to get in contact with the intracellular matrix of the plant cell (e.g., in leaves) and endogenous electron donors become available for LPMOs, such as ascorbic acid or reduced glutathione (Figure 4). Moreover, it is likely that photosynthetic pigments, such as chlorophyll, are released from the chloroplast during the decay of the plant cell and donate electrons for LPMOs. Based on the current knowledge, however, it remains debatable to what extent the latter system will contribute to the LPMO-assisted PCW degradation *in vivo*.

Catalases and superoxide dismutases are secreted by fungi and have been shown to potentially decrease the inactivation of LPMOs via the regulation of the reactive oxygen species concentration (Zintel et al., 2011; Schneider et al., 2016). However, the role of these enzymes during the LPMO-mediated plant biomass degradation is currently challenged due to a not fully understood reaction mechanism, in particular, regarding the role of O_2_ or H_2_O_2_ during the catalytic cleavage of the substrate (Phillips et al., 2011; Scott et al., 2016; Walton and Davies, 2016; Bissaro et al., 2017; Möllers et al., 2017; Simmons et al., 2017; Hangasky et al., 2018).

## Future prospects

### Substrate specificity and C1/C4-regioselectivity

First described to oxidize crystalline polysaccharides like chitin and cellulose, it became apparent that LPMOs oxidize a much wider range of substrates including various hemicelluloses. At this moment it is not clear which of the above-mentioned substrate specificities of LPMOs are most beneficial to improve the activity of hydrolytic enzyme cocktails toward plant biomass. Presumably, the decomposition of diverse PCW structures demands the presence of LPMOs that comprise diverse substrate specificities like hemicellulose-active LPMOs, but the role of these LPMOs during plant biomass degradation has still to be proven experimentally. Furthermore, we do not know why fungi express LPMOs that oxidize soluble oligosaccharides since hydrolytic enzymes such as cellobiohydrolases can degrade these substrates significantly faster. Certainly, LPMOs that oxidize crystalline polysaccharides are able to enhance the substrate accessibility and improve the activity of hydrolytic enzymes (Vaaje-Kolstad et al., 2005a; Harris et al., 2010). At this moment, it remains enigmatic why chitin-oxidizing LPMOs boost the activity of chitinases toward chitin to a larger extent than cellulose-oxidizing LPMOs added to cellulases in the presence of cellulose (Vaaje-Kolstad et al., 2010; Forsberg et al., 2011, 2014a). Moreover, we suggested that LPMOs may comprise further mode of actions, like processive or random polysaccharide-oxidizing LPMOs, or, as already shown, LPMOs that preferably cleave amorphous cellulose rather than crystalline cellulose (Villares et al., 2017; Table 1). But, how the mode of action of LPMOs affects plant biomass degradation and the activity of hydrolytic enzymes remains to be answered.

Methods like proteomics, in particular, analysis of secretomes or fungi grown on different substrates have a good potential for the identification of LPMOs with promising substrate specificities. These techniques led to the recent discovery of the AA14 family by studying the growth of the white-rot fungus *Pycnoporus coccineus* in the presence of different types of biomass (Tian et al., 2009; Berka et al., 2011; Couturier et al., 2018). These findings about various substrate specificities and C1/C4-regioselectivities of LPMOs will contribute to the development of more “tailor-made” enzyme cocktails and evoke possibilities for new applications, such as polysaccharide modification to form novel polysaccharides.

### Reducing agent specificity

Several electron-donating systems of LPMOs have been described so far, but it can only be speculated which system is actually of relevance for different types of fungi that comprise certain modes of life-style, such as saprophytes and plant pathogenic fungi. Further investigation of these systems would potentially provide more answers concerning the role of electron-donating systems during plant biomass degradation, especially when aiming to improve the activity of hydrolytic enzyme cocktails comprising LPMOs (Westereng et al., 2015; Frommhagen et al., 2016). Interestingly, lignin is the most abundant plant polyphenol in biomass. However, only limited amount of work has been done on the interaction between lignin (e.g., high and low molecular weight lignin; H, G, and S units) and LPMOs and its role during LPMO-mediated plant biomass degradation, which merits future investigation (Westereng et al., 2015). In addition, Hu et al. (2014) demonstrated that pretreatments affect the cellulose hydrolysis and thereby also the extent of the LPMO-mediated boosting effect. Therefore, optimization of the plant biomass degradation with LPMO-enriched enzyme cocktails and the simultaneous development of pretreatments should include the focus on the potential of residual lignin as a reducing agent.

Most of the commercial enzyme cocktails that have been recently developed are enriched with LPMOs to varying amounts, like Cellic CTec2 and CTec3 (Chylenski et al., 2017). The increase of the hydrolytic activity that can be achieved by the addition of AA9 LPMOs to these enzyme cocktails varies, depending on the type of the LPMO and the substrate that has been used for the activity assay (Merino and Cherry, 2007; Hu et al., 2014, 2015). Notably, it should be considered that the addition of LPMOs to an enzyme cocktail is limited due to the increased formation of oxidized moieties that are known inhibitors of hydrolases, like β-glucosidases. In addition, these oxidized moieties also inhibit subsequent ethanol fermentation since only a limited amount of these compounds can be metabolized by *Saccharomyces cerevisiae* species (Peinado et al., 2003, 2006; Cannella et al., 2012).

### Catalytic mechanism and activity

Vaaje-Kolstad et al. (2010) presented LPMOs as monooxygenases that demand molecular oxygen and external electron donors to oxidize polysaccharides (Vaaje-Kolstad et al., 2010). This widely accepted catalytic mechanism has recently been expanded by findings of Bissaro et al. (2017), which indicate that hydrogen peroxide-fueled LPMOs are able to act under anaerobic conditions (Bissaro et al., 2017). This finding is of high importance for future applications, since biomass degradation at an industrial scale is mainly conducted under oxygen-poor conditions. A changed view on the catalytic mechanism will also improve the understanding of how LPMOs are effected by different electron-donating systems in the presence of different substrates (Simmons et al., 2017). Further investigation should aim at the improved understanding of the structure-function relationships of LPMOs, which would also pave the way for protein engineering methods, such as directed evolution (Forsberg et al., 2017). These methods should initially aim at the improvement of the LPMO's operational stability, rather than the activity of the enzyme (Bissaro et al., 2017). The characterization of LPMOs should be expended by the investigation of the effect of varying incubation conditions on LPMO activity, especially in the presence of different reducing agents. This research is of high importance from an application point of view, since pH and temperature influence the catalytic performance of LPMOs significantly (Frommhagen et al., 2017c).

## Concluding remarks

LPMOs have initially been shown to oxidize hardly accessible substrates, however, it has become apparent that these powerful enzymes cleave a much broader range of PCW polysaccharides in the presence of various electron-donating systems. The discovery of new potential substrates and electron-donating systems of LPMOs is a continuous process, whereas the role of these enzymes during PCW degradation and plant-pathogen interactions has hardly been explored so far. Recent discoveries of new AA families, in particular AA14 and AA15, show the diversity of LPMO sources, ranging from bacteria, fungi, viruses and insects, which illustrate how widely these enzymes are distributed throughout nature. Here, we summarized findings obtained from *in vitro* studies of LPMO substrate-specificities and electron-donating systems to emphasize their potential role for fungi *in vitro*. The latter is expected to contribute to the further understanding of these enzymes and, possibly, will promote new possibilities for a better usage of LPMOs within the biotechnology field.

## Author contributions

MF designed and wrote the manuscript. AW and MF built the LPMO models. MK and WvB contributed to the design and performed the critical revision of the manuscript. All authors read and approved the final manuscript.

### Conflict of interest statement

The authors declare that the research was conducted in the absence of any commercial or financial relationships that could be construed as a potential conflict of interest.
